# Correction to ‘Frailty determinants in heart failure: inflammatory markers, cognitive impairment and psychosocial interaction’

**DOI:** 10.1002/ehf2.15428

**Published:** 2025-09-16

Wleklik, M. , Lee, C. S. , Lewandowski, Ł. , Czapla, M. , Jędrzejczyk, M. , Aldossary, H. , and Uchmanowicz, I. Frailty determinants in heart failure: inflammatory markers, cognitive impairment and psychosocial interaction. ESC Heart Failure, 2025;12:2010–2022. 10.1002/ehf2.15208 39853613 PMC12055405

In the ‘Funding’ section, the current statement:

‘This research was funded by the National Science Centre, Poland [Narodowe Centrum Nauki (NCN), Grant OPUS 22 OPUS.E250.22.003].’

should be replaced with

‘This research was funded by the National Science Centre, Poland No: 2021/41/B/NZ7/01698.’

We apologize for this error.

